# Arthroscopic capsular release for idiopathic frozen shoulder with intra-articular injection and a controlled manipulation

**DOI:** 10.1308/003588414X13824511650452

**Published:** 2014-01

**Authors:** CD Smith, P Hamer, TD Bunker

**Affiliations:** Royal Devon and Exeter NHS Foundation Trust,UK

**Keywords:** Frozen shoulder, Adhesive capsulitis, Oxford shoulder score, Range of movement

## Abstract

**INTRODUCTION:**

The aim of this prospective study was to assess the immediate and long-term effectiveness of arthroscopic capsular release in a large cohort of patients with a precise and isolated diagnosis of stage II idiopathic frozen shoulder.

**METHODS:**

All patients underwent a preoperative evaluation. Patients with secondary frozen shoulder and those with concurrent pathology at arthroscopy were excluded. This left 136 patients with a stage II arthroscopically confirmed idiopathic frozen shoulder. At each postoperative attendance, a record was made of pain, function and range of motion. At 12 months, the Oxford shoulder score was calculated, and pain and range of motion were assessed.

**RESULTS:**

Fifty per cent achieved good pain relief within a week and eighty per cent within six weeks of arthroscopic capsular release. The mean preoperative visual analogue scale pain score was 6.6 and the mean postoperative score was 1.0. The mean time to achieving good pain relief was 16 days following surgery. No patient could sleep through the night prior to surgery while 90% reported having a complete night’s sleep at a mean of 12 days after surgery. The mean postoperative Oxford shoulder score was 38/48 and the mean improvement was 19.2.

**CONCLUSIONS:**

This large series demonstrates that arthroscopic capsular release is a safe procedure, with rapid improvement in pain and a marked improvement in range of motion.

The term frozen shoulder was first used in 1934 by Codman, who described the common features of a slow onset of pain felt near the insertion of the deltoid muscle, inability to sleep on the affected side, and restriction in both active and passive elevation and external rotation, yet with a normal radiological appearance.^1^ The pathology of frozen shoulder involves active fibroblastic proliferation in the capsule of the shoulder joint, accompanied by some transformation of fibroblasts to myofibroblasts.^2,3^ Despite this knowledge of the pathology, there is no consensus on the most favourable method of managing the disease. Suggestions for management range from supervised neglect to corticosteroids, physiotherapy, manipulation, hydrodilatation and arthroscopic capsular release.^4^ A review of the literature presents seven studies that discretely present data for arthroscopic capsular release in primary frozen shoulder (Table 1).^5–11^

**Table 1:** Previous studies in the literature on the outcome of arthroscopic capsular release

Study	Number of patients	Follow-up duration	Result of primary outcome measure
Warner^5^	23	24–64 months	Mean improvement in CS: 48 points
Jerosch^6^	28	6–48 months	Mean improvement in CS: 41 points
Berghs^7^	25	3–40 months	Mean improvement in CS: 50 points
Baums^8^	30	24–72 months	Mean improvement in ASES: 56 points
Cinar^9^	28	13–99 months	Mean improvement in CS: 64 points
Elhassan^10^	41	26–75 months	Mean improvement in CS: 55 points
Waszczykowski^11^	14	Minimum 24 months	Mean improvement in ASES: 74 points

CS = Constant-Murley score; ASES = American Shoulder and Elbow Surgeons score

The sample size of these studies is very small: one study had just 14 patients^11^ and only one study had more than 30 patients.^10^ All of the studies contain results at the two-year mark and four contain results at over five years.^5,8–11^

It is difficult to form rational decisions on treatment because many of the studies lack exclusion criteria (eg stiffness secondary to trauma, previous surgery, rotator cuff disease, radiotherapy); lack inclusion criteria (arthroscopically proven stage II idiopathic frozen shoulder,^12^ with exclusion of other disease by arthroscopy); have small sample sizes; use unvalidated outcome measures; and do not report early results.

What the patient with frozen shoulder wants is immediate and long lasting benefit from intervention. The purpose of this study was to determine the immediate and long-term benefit, in terms of pain relief and improvement in function, of arthroscopic capsular release for idiopathic frozen shoulder.

## Methods

A total of 172 patients were entered prospectively into a patient reported outcome study starting in November 2005 and ending in May 2010. The initial evaluation included completion of the Oxford shoulder score (OSS) questionnaire, a detailed history and a standardised examination with a record of range of motion.

Twenty-two patients were excluded as they had stiffness due to previous open or closed shoulder surgery, fractures or radiotherapy. This left 150 patients with a clinical diagnosis of stage II idiopathic frozen shoulder.^12^ Three senior clinicians, each with 10–25 years of experience of shoulder surgery, made the diagnosis. The diagnosis was based on Codman’s clinical criteria^1^ and the American Shoulder and Elbow Surgeons (ASES) consensus definition of insidious onset of true shoulder pain, with night pain and restriction of passive forward elevation to less than 100º and external rotation less than one half of normal.^13^ The indication for surgery was night pain that disturbed the patient’s sleep most or every night, regardless of the duration of symptoms or non-operative management. Patients had symptoms present for a mean of 11 months (standard deviation [SD]: 7 months) and a minimum of 2 months prior to operation.

We elected to include diabetic patients in the study as previous histological studies had shown no difference in the pathology of the capsule between diabetics and non-diabetics.^2^ However, it was agreed that the results would be broken down at the end of the study to compare early and late results between diabetics and non-diabetics. All patients were in stage II of the disease (ie they had marked stiffness as well as severe pain).^12^ Any patient beginning the resolution phase was excluded automatically from intervention. Sixty-two per cent of our patients had already had an injection of steroid and had failed to respond.

All patients underwent a standardised shoulder arthroscopy by the same surgeon. At arthroscopy, a further 14 patients with rotator cuff disease (partial or full-thickness tear) or early arthritis undetected previously by radiography were excluded. This left 136 patients with a decreased capsular volume, angiogenesis, a thickened capsule and rotator interval filled with fibrotic tissue who underwent arthroscopic capsular release. These patients were followed up at three weeks after surgery as well as at two and three months. Pain, function and range of motion were recorded at each attendance. The long-term outcome evaluation included the OSS, assessment of pain and range of motion.

The arthroscopic capsular release was performed by the same surgeon throughout the study period under scalene blockade and light general anaesthesia. The arthroscopic findings were recorded in visual and written form in the notes, along with a record of the releases performed and the range of motion achieved in every plane. Multiple arthroscopic photographs were taken of each case and kept in the notes.

The release started with a synovectomy of the rotator interval. Subsequently, diathermy excision of the rotator interval was performed until the extracapsular fat and the deep surface of the coracoid were exposed. An assessment was then made of the subscapularis tendon, the overlying scar tissue and the middle glenohumeral ligament. The scar tissue and middle glenohumeral ligament lying over the subscapularis were excised until the normal transverse shining fibres of the top surface of the subscapularis were exposed. The superior tendon of the subscapularis was never sacrificed. This release was continued down to the five o’clock position. Attention was then switched back to the interval and the capsule behind the long head of the biceps (LHB) was excised, up until the front edge of the supraspinatus was seen. The LHB was preserved in all patients.

The diathermy probe was now passed behind the LHB, and the superior and posterosuperior capsule was divided, parallel to the joint surface, on the outer edge of the labrum, until the muscular fibres of the supraspinatus were exposed. This release was continued until the posterior portal was reached. The arthroscope was then removed and the range of motion checked. If still quite stiff, the arthroscope was reintroduced and, using a switch stick, the posterior capsule was released. Finally, 80mg of methylprednisolone and 10ml of 0.5% bupivacaine were injected down the arthroscope cannula into the joint, and a gentle manipulation completed the inferior release. Each arthroscopy was performed with good fluid input and output as well as intermittent diathermy to ensure there was no risk of chondrolysis.

All patients were instructed in Codman exercises^1^ and attended physiotherapy, where treatment was monitored and recorded. Patients were seen by a clinician at three weeks, when the range of motion, the level of pain and night pain were recorded. Patients were then seen monthly up until three months. A final OSS questionnaire was sent to all patients after 12 months along with a brief questionnaire about their treatment experience. A total of 136 patients were contacted and 101 returned the questionnaire.

An unpaired Student’s t-test was used for two-way analysis of variance. Correlation between data sets was analysed using Pearson’s correlation coefficient. Frequency data were analysed using a 2×2 contingency square table and tested with Pearson’s uncorrected test. Statistical significance was set at an alpha level of 0.05.

## Results

The mean age at the time of surgery was 52 years (range: 34–72 years, SD: 7.5 years). Sixty-four patients were female. Fifty-five patients had surgery performed on the left shoulder and forty-six the right. Forty-five patients had surgery on the dominant arm. Twenty-three patients were diabetic.

### Pain

**Figure 1:**
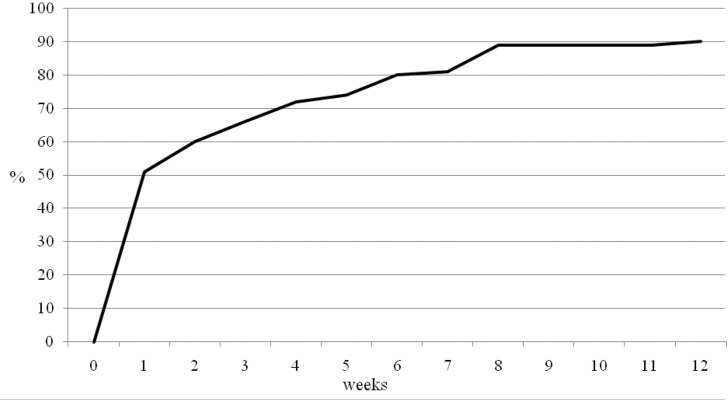
Summative time to pain relief

All patients complained of lateral upper arm pain prior to surgery. Ninety per cent felt that their pain had been relieved significantly by the surgery. Over half (51%) stated that their pain was reduced significantly in the first postoperative week. By week 6, 80% stated that they had good pain relief and at three months, 90% had good pain relief. However, 10% of patients continued to have some pain despite the intervention (Fig 1).

The mean preoperative visual analogue scale (VAS) pain score was 6.6 (SD: 2.4) (no pain = 0, severe pain = 10). The postoperative pain score was 1.0 (SD: 2.0). The mean improvement in scores was 5.4 (SD: 2.8). There was no statistical difference in the preoperative and postoperative VAS scores between the diabetics and the non-diabetics (*p*=0.37 and *p*=0.21 respectively).

Ten patients failed to get pain relief from the intervention, yet nine of these stated that they would recommend it to a friend. Thus, 99% of the total cohort of patients said they would recommend this form of treatment to a friend with proven stage II idiopathic frozen shoulder.

**Figure 2:**
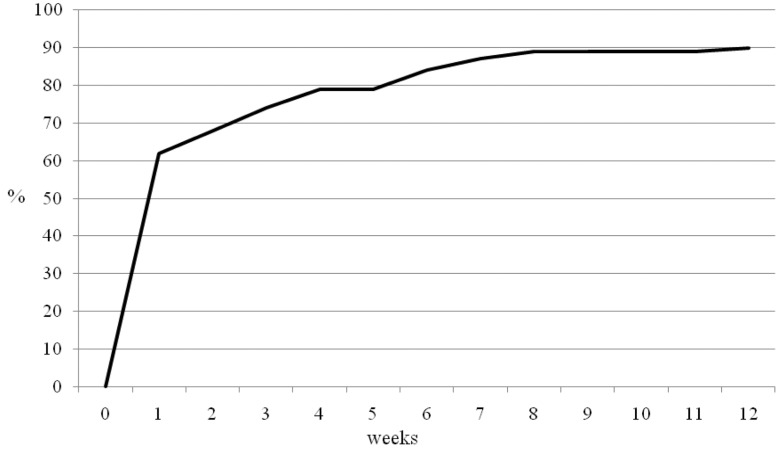
Summative time to be able to sleep through the night

Ninety per cent of patients stated that the surgery allowed them to have an uninterrupted night’s sleep (Fig 2). All 136 patients were unable to sleep through the night preoperatively. However, 62% had an uninterrupted night’s sleep by week 1 following intervention and 84% by week 6. Ten patients continued to have night disturbance despite the intervention.

Thirty-nine per cent of patients complained of preoperative pain that radiated below the elbow and 25% complained of ‘pins and needles’ in the arm or hand prior to surgery. All except one patient had complete resolution of the pain below the elbow and ‘pins and needles’ after surgery.

### Range of movement

**Figure 3:**
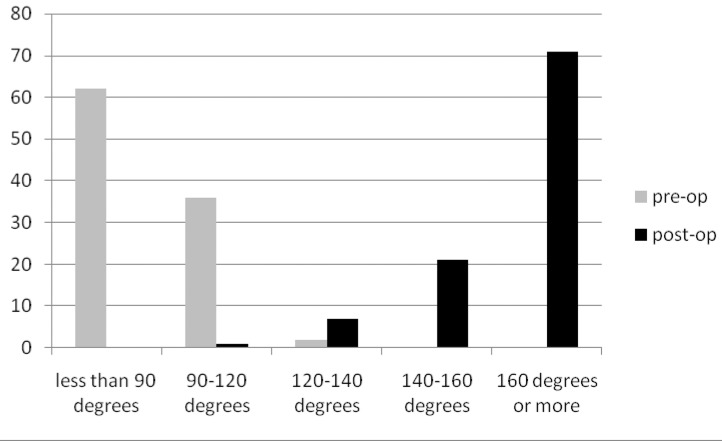
Percentage of patients and range of forward fl exion

**Figure 4:**
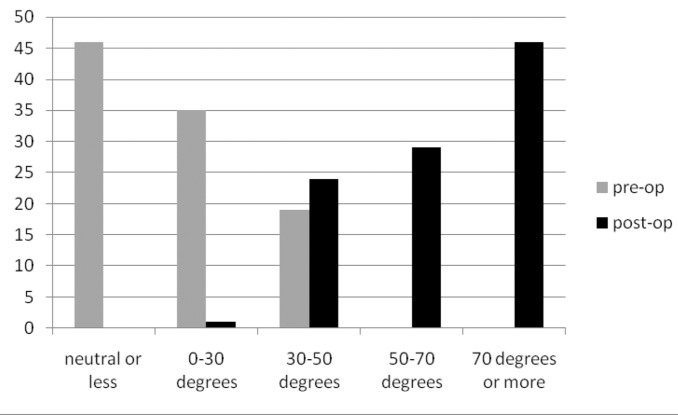
Percentage of patients and range of external rotation

**Figure 5:**
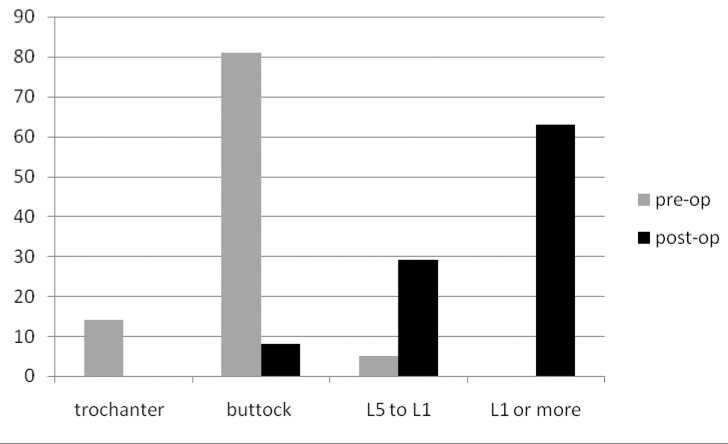
Percentage of patients and range of internal rotation

The preoperative and postoperative range of movements are presented in Figures 3–5. External rotation was measured with the arm adducted. In the diabetic group, only 48% had regained forward flexion greater than 160º, 30% had regained internal rotation to L1 or greater and 17% had regained external rotation greater than 70º. This compared with 79%, 73% and 55% respectively in the non-diabetic group (*p*<0.01).

### Long-term questionnaire

Seventeen per cent of patients complained they had lost some of the original gain in range of movement following surgery. Seven patients stiffened to the extent that they felt the surgery was not successful. There was no significance of age, sex, preoperative movement, duration of symptoms, preoperative OSS or diabetes in this ‘failure’ group. Despite the failure, six of these seven patients were still appreciative of their treatment.

### Oxford shoulder score

The mean pre- and postoperative scores are shown in Table 2. There was no significant difference in preoperative OSS (*p*=0.74) and postoperative OSS (*p*=0.25) or gain in OSS (*p*=0.43) between the diabetic group and the non-diabetic group. There was also no correlation between postoperative OSS and duration of symptoms prior to surgery (r=-0.07). One patient had a worse pain score after the procedure and the breakdown of the OSS demonstrates a better improvement in pain scores than functional scores. However, 48% still did not score full points on the pain element of the OSS.

**Table 2:** Mean pre- and postoperative Oxford shoulder scores

	Pain(best = 16)	SD	Function(best = 32)	SD	Total score(best = 48)	SD
Preoperative	3.0	2.1	15.9	6.2	19.2	7.4
Postoperative	12.9	4.1	25.2	4.9	38.1	8.6
Mean change	9.8	4.3	10.0	6.0	19.2	10.2

SD = standard deviation

### Function

**Figure 6:**
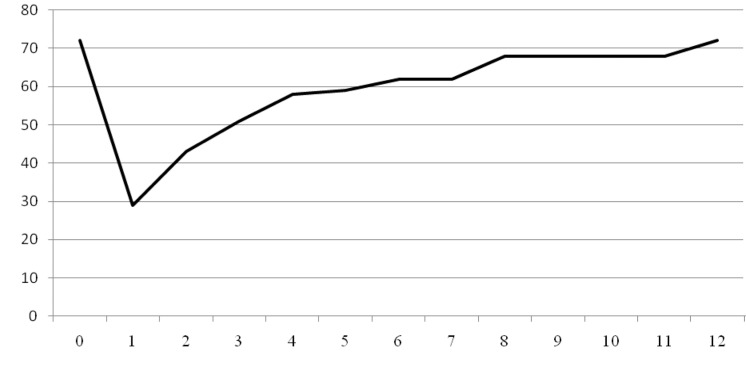
Summative time to return to work

Seventy-four per cent of the patients were in work prior to surgery. The rest were retired (15%) or unemployed (11%). All patients who worked prior to the intervention returned to work. Thirty-nine per cent returned in week 1, fifty-eight per cent by week 2 and sixty-nine per cent by week 3 (Fig 6) although each patient was advised to avoid work for three weeks. Six patients were off work for more than six weeks but all had returned to work by three months.

### Complications

One patient had an infected portal site that responded to antibiotics. There was no deep infection or chondrolysis. There was no episode of instability or dislocation. There was no nerve injury.

### Contralateral symptoms

In the follow-up questionnaire, 53% of the patients described having had symptoms of frozen shoulder in the contralateral shoulder at some point since the time of operation and 32% had required treatment for frozen shoulder in the contralateral shoulder. There was no difference in OSS between patients with eventual contralateral symptoms and those without (*p*=0.83).

## Discussion

The natural history of frozen shoulder is ill understood but is not as benign as Codman suggested in 1934.^1^ Two studies have shown residual symptoms in 50% of patients after an average of five and seven years in patients treated non-operatively.^14,15^

This study has identified patients with arthroscopically proven frozen shoulder. The clinical diagnosis of frozen shoulder can be imprecise as other conditions such as mild arthritis and stiffness secondary to rotator cuff disease can be a mimic. These secondary causes can only be excluded by arthroscopy (even magnetic resonance imaging will not show early arthritis). In the present study, a clinical diagnosis made by highly experienced shoulder surgeons was shown to be wrong at arthroscopy in 14 patients (9%), who were then excluded. Other studies have demonstrated that patients with a clinical diagnosis of frozen shoulder are found to have associated pathology over 40% of the time.^16^ It is likely that all non-surgical series will contain a similar error.

This study only included those patients who met Codman’s criteria for frozen shoulder with night pain that disturbed their sleep most or every night and many had received steroid injections without benefit prior to review in the specialist shoulder clinic. Consequently, this reflects those patients with significant symptoms and the indication for intervention was severe pain that prevents sleep. The study excluded patients with a secondary cause of frozen shoulder, which behaves in a different manner to idiopathic frozen shoulder.

A serious failing of most studies is sample size. It has been suggested that studies into frozen shoulder should state the exact phase of the patients being enrolled and have a precise diagnosis.^17^ It has also been proposed that future research should target interventions performed during the painful stage of frozen shoulder.^18^ Our study is the first with over 100 patients undergoing arthroscopic capsular release for isolated proven stage II (severe pain and stiffness) idiopathic frozen shoulder.

This study used the OSS, a validated outcome score.^19^ The results suggest there is a discrepancy between the patient’s perception of success and patient reported function and pain scores. It also indicates that patients can tolerate some residual stiffness and some residual pain but that what they really appreciate is the abolishment or reduction in the severe pain that prevents sleep. Night pain is a key component of frozen shoulder and the main indication for intervention. However, few studies report on recovery from night pain. In this study, 90% of patients stated that the surgery allowed them to have an uninterrupted night’s sleep. All 136 patients were unable to sleep through the night preoperatively. By week 1, 60% had an uninterrupted night’s sleep following intervention and 80% by week 6.

The OSS showed similar outcomes in diabetics and nondiabetics, yet objectively the diabetics had stiffer joints at outcome. This is likely to be due to the OSS reflecting functional range rather than extreme range. This loss of end range might not be important to elderly inactive patients but it might be important to active 50-year-old individuals.

Finally, the purpose of any intervention in stage II idiopathic frozen shoulder must be to shorten the natural history of the disease process. For an intervention to have any use it must show rapid improvement (pain free, sleeping and moving within 1–2 weeks) and there is no purpose in demonstrating improvement at one year’s follow-up in a condition where the natural history is for subjective improvement in at least 50% of patients without any treatment.^20,12^ This study looked at early improvement and whether the early improvement was sustained. The majority (90%) of patients felt that their pain had been relieved by the surgery. Half (50%) stated that their pain had gone in the first postoperative week. By week 6, 80% stated their pain had gone.

One previous small study that included both secondary and primary frozen shoulder did demonstrate a significant reduction in pain at one week, maintained to all time points.^22^ This present study adds further weight to this rapid improvement.

Some might suggest that injecting 80mg of prednisolone into the joint at the end of the release could be responsible for the rapid improvement in our study. However, nearly two-thirds of the patients had already been given an intra-articular injection without any benefit.

Assessing the impact of intra-articular injection of steroids in the literature is difficult for the reasons stated above (inaccurate diagnosis, exclusions, inclusions, sample size, follow-up and lack of control groups). Improvements in pain, ASES and SF-36^®^ scores, and movement have been demonstrated with intra-articular steroids as a sole treatment at four weeks.^23^ Nevertheless, a controlled study of intra-articular injection of steroids, physiotherapy, placebo and injection of saline showed that at 6 weeks the steroid group had a measurable improvement in pain but no improvement in movement compared with the placebo group, and by 16 weeks there was no demonstrable difference between steroid injection and placebo.^24^ Intra-articular injections have also been shown to give equal benefit compared with manipulation but examination of these results shows that improvement is measured in months rather than days.^25^

It is likely that the installation of prednisolone had a beneficial effect on pain. Nevertheless, it is unlikely that the rapid benefit seen was all due to the steroid.

Interestingly, ten patients failed to get pain relief, yet nine said they would still recommend the procedure. This may be because all patients achieved some increase in range of movement and all except for one patient had some improvement in the VAS pain scores.

Despite rapid early improvement in range of motion and function, 17% reported that a degree of stiffness returned during the rehabilitation phase. We attempted to see if there were any factors associated with poor outcome. There was no significant difference in the sex, age, preoperative range of movement, preoperative pain score, duration of symptoms prior to surgery, preoperative OSS or the incidence of diabetes between those patients with stiffness, who thought their operation had not been a success, and the rest of the index group.

Contralateral involvement has been quoted in the literature as between 6% and 34%.^3,26,27^ This study, however, would suggest that contralateral involvement may be even higher than thought previously.

Over a third of the patients had complained of pain that radiates below the elbow and a quarter had ‘pins and needles’ in the forearm or hand prior to surgery, both of which resolved following surgery. This has not been documented previously.

The study can be criticised for using a questionnaire to record return to work if this was not recorded in the contemporaneous notes. Recall bias is known to have a significant impact on the validity of outcomes.^28^ However, return to work is a major life event after surgery and is probably recalled reasonably accurately. Despite this, these results should be interpreted with caution. This study would have benefited from an OSS in the first month. On the positive side, the study was a prospective audit of the early and late outcomes of arthroscopic capsular release in over 100 patients with stage II idiopathic frozen shoulder, using proper exclusion criteria, an accurate diagnosis confirmed by shoulder arthroscopy, with a validated preoperative and postoperative functional outcome score.

## Conclusions

This large study with strict inclusion and exclusion criteria has demonstrated that arthroscopic capsular release with intra-articular steroid injection and a controlled manipulation has a rapid impact on the pain, night pain and range of motion of stage II idiopathic frozen shoulder. By week 1, 50% of patients had marked pain relief and 60% had uninterrupted sleep. There was a marked improvement in function by week 3: 39% returned to work at week 1 and 58% by week 2. There was one portal site infection that settled rapidly with antibiotics. There was no deep infection; no instability or dislocation, no nerve injury and no chondrolysis. This is an effective procedure with minimal complications.
